# *α*-Lipoic Acid Targeting PDK1/NRF2 Axis Contributes to the Apoptosis Effect of Lung Cancer Cells

**DOI:** 10.1155/2021/6633419

**Published:** 2021-06-04

**Authors:** Liduo Yue, Yanbei Ren, Qingxi Yue, Zhou Ding, Kai Wang, Tiansheng Zheng, Guojie Chen, Xiangyun Chen, Ming Li, Lihong Fan

**Affiliations:** ^1^Department of Respiratory Medicine, Shanghai Tenth People's Hospital, Tongji University School of Medicine, 200072 Shanghai, China; ^2^Department of Oncology, Shanghai 9th People's Hospital, Shanghai Jiao Tong University School of Medicine, 201999 Shanghai, China

## Abstract

As an antioxidant, *α*-lipoic acid (LA) has attracted much attention to cancer research. However, the exact mechanism of LA in cancer progression control and prevention remains to be unclear. In this study, we demonstrated that *α*-lipoic acid has inhibitory effects on the proliferation, migration, and proapoptotic effects of non-small-cell lung cancer (NSCLC) cell lines A549 and PC9. LA-induced NSCLC cell apoptosis was mediated by elevated mitochondrial reactive oxygen species (ROS). Further study confirmed that it is by downregulating the expression of PDK1 (the PDH kinase), resulted in less phospho-PDH phenotype which could interact with Keap1, the negative controller of NRF2, directly leading to NRF2 decrease. Thus, by downregulating the NRF2 antioxidant system, LA plays a role in promoting apoptosis through the ROS signaling pathway. Moreover, LA could enhance other PDK inhibitors with the proapoptosis effect. In summary, our study shows that LA promotes apoptosis and exerts its antitumor activity against lung cancer by regulating mitochondrial energy metabolism enzyme-related antioxidative stress system. Administration of LA to the tumor-bearing animal model further supported the antitumor effect of LA. These findings provided new ideas for the clinical application of LA in the field of cancer therapy.

## 1. Introduction

Cancer cells exhibit increased ROS levels, partly due to oncogenic stimulation compared to normal cells, increasing their metabolic activity [1–3]. On the one hand, ROS could increase the membrane potential, leading to changes in the membrane permeability, release of cytochrome C, and inducing apoptosis [4]. On the other hand, the cancer cells could upregulate nuclear factor erythroid 2-related factor 2 (NRF2), the antioxidant system to inhibit the apoptotic pathway [5]. Under persistent oxidative stress, cancer cells become well adapted to such stress through a set of mechanisms that activate ROS-scavenging systems, including NRF2-Keap1-ARE system, and inhibit apoptosis [6].

Proteasomes rapidly degrade the nuclear factor erythroid 2-related factor 2 (NRF2) under a basal condition in a Keap1-dependent manner. ROS oxidatively modifies Keap1 to release NRF2 and allow its nuclear translocation [7]. It then binds to the antioxidant response element to regulate downstream gene transcription. The NRF2 antioxidant system is the key to oxidative stress, maintaining redox homeostasis [8–10]. With increasing oxidative stress, the NRF2 is highly expressed, antioxidant proteins are upregulated, and then the cells escape from apoptosis. Meanwhile, higher NRF2 constantly interacts with the surrounding immune environment, resulting in the proliferation of cancer cells, immune escape, and malignant transformation [11, 12]. Several lines of evidence suggest that NRF2 is involved in cancer development and recurrence and resistance to anticancer drugs [9, 13, 14]. Therefore, understanding the mechanisms of ROS regulation is essential to kill cancer cells and overcome drug resistance efficiently.

*α*-Lipoic acid (LA) is a substance containing disulfide, known as 1,2,3-disulfide heterocyclic pentane pentanoic acid [15]. It is synthesized in the mitochondria. LA is a natural coenzyme in the mitochondrial pyruvate dehydrogenase (PDH) complex. PDH complex catalyzes the oxidative decarboxylation of *α*-keto acids, such as pyruvate and *α*-ketoglutarate [16]. It also regulates various metabolic enzymes in glucose catabolism [17]. LA forms a covalent bond with the side chain of a lysine residue within the E2 subunit of the PDH complex and promotes the enzyme activity of pyruvate dehydrogenase (PDH) [18]. PDH complex is the critical switch from glycolysis to oxidative phosphorylation (OXPHOS). Also, LA has been reported to promote apoptosis in several cancer subtypes [19–21]. It can induce ROS production and lead to cancer apoptosis [22, 23]. However, the molecular mechanism still remains to be unclear. Hence, in this study, the underlying relationship between the switching of glycolysis to OXPHOS and proapoptosis of LA was investigated.

Finally, based on the widely accepted theory of the Nobel Prize winner Warburg, “the tumor is a kind of metabolic disease,” most lung cancer relies on glycolysis to generate energy, including the big lung cancer [24]. Because PDK1 suppresses the switch of glycolysis to OXPHOS by phosphorylation of Ser 293 of PDH [25], it has been regarded as an anticancer target [26, 27]. Nevertheless, the clinical effect and application still need to be evaluated. The NSCLC cell lines A549 and PC9 have been reported to prefer glycolysis for energy supply [28]. So, we test if LA could influence the metabolic feature and directly regulate the antioxidant system to promote apoptosis on these two cell lines.

## 2. Result

### 2.1. LA Inhibits Proliferation and Induces Apoptosis in A549 and PC9 Lung Cancer Cells

**Figure 1:**
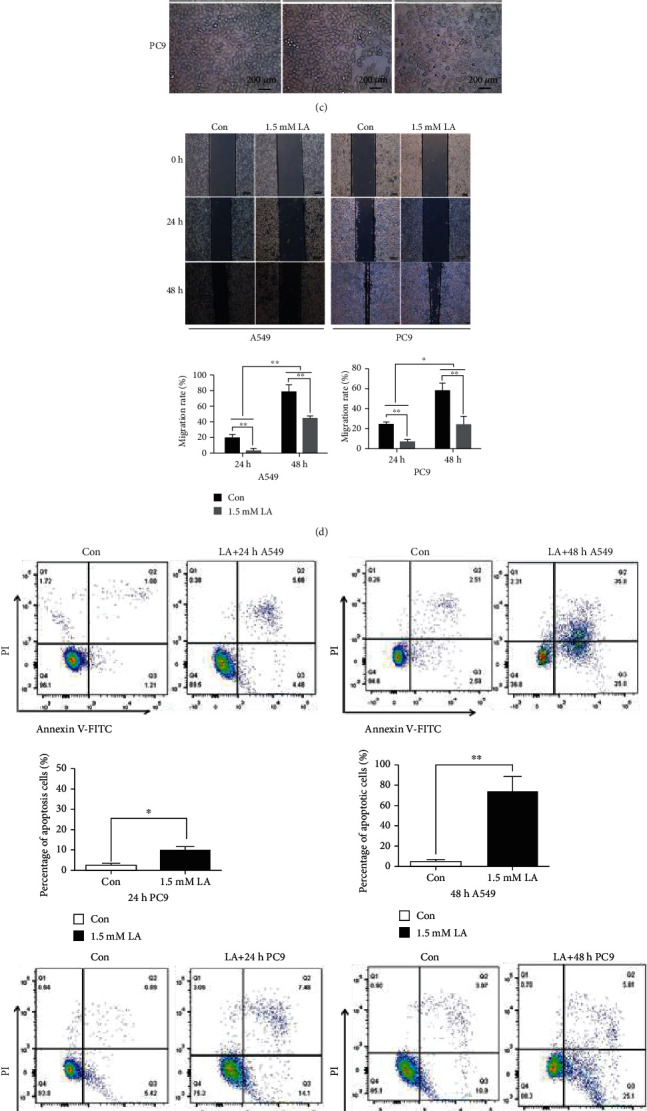
LA inhibits proliferation, induces apoptosis, and inhibits shift rate in lung cancer cells. (a) Chemical structure of LA. (b) Lung cancer cell lines were treated with different doses of LA for 24 h or 48 h. Cell proliferation was examined by CCK8 assay, and the IC50 values of LA were calculated by GraphPad (Prism) software. (c) Cell morphology were captured under the bright field of microscope exposed to different doses of LA. (d) LA inhibits the shift of A549and PC9 cells in a time-dependent manner. (e) The cells were exposed to 1.5 mM of LA for 24 h and 48 h, and then, Annexin/PI double staining was used to evaluate the apoptotic rates. Results were shown as the mean ± s.d. The symbol ∗ indicates *P* < 0.05; ∗∗ indicates *P* < 0.01.

To explore the antitumor effects of LA (Figure 1(a)) in lung cancer cells, A549 and PC9 lung cancer cell lines were treated with different concentrations of LA for 24 h or 48 h, respectively. Cell proliferation was evaluated by CCK8 assay (Figure 1(b)). The results showed that LA could inhibit the proliferation of lung cancer cells A549 and PC9 with IC50 at 3-6 mM. Under the bright-field view of the cells that are processed with different concentrations of LA, 1.5 mM LA showed noticeable apoptotic features (cell becomes longer) but not much cell death (Figure 1(c)). 1.5 mM LA could also effectively inhibit the shift of lung cancer cells (Figure 1(d)), coinciding with the antiproliferative effects. So, 1.5 mM LA was used for testing the mechanism of apoptosis in this work. A549 and PC9 cells were exposed to 1.5 mM LA for 24 h and 48 h. LA treatment increased the percentage of apoptotic cells (Figure 1(e)). Moreover, the longer the time of LA incubation, the more pronounced was the apoptosis.

### 2.2. LA Promotes Apoptosis of Lung Cancer Cells by Elevating ROS Signaling

**Figure 2:**
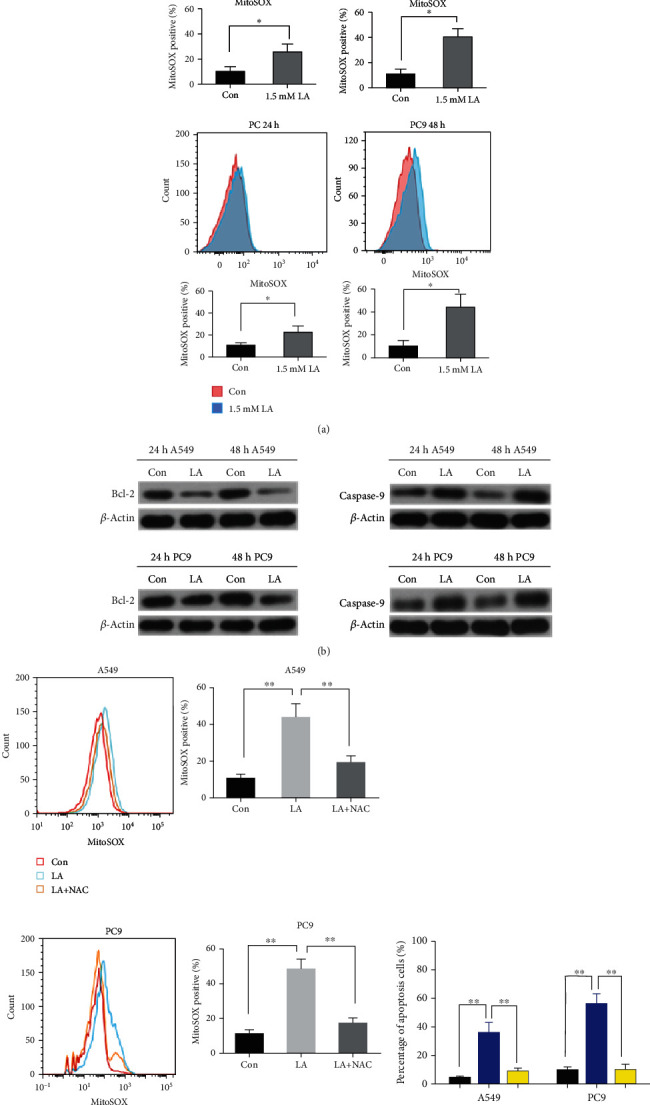
LA can induce mitochondrial ROS level of lung cancer cells. (a) The cells were incubated with 1.5 mM LA for 24 h and 48 h, and the mitochondria ROS was measured using MitoSOX red probe. (b) After incubation with 1.5 mM LA for 24 h and 48 h, the apoptotic proteins Bcl-2 and caspase-9 were examined by western blotting. (c) NAC quenched the ROS induced by LA. (d) NAC abbreviated the apoptotic effect induced by LA. Results are shown as the mean ± s.d.∗ indicates *P* < 0.05; ∗∗ indicates *P* < 0.01.

Using the MitoSOX red probe to label the mitochondrial ROS, we confirmed that the ROS of the two lung cancer cell were remarkably increased after exposed to LA. Moreover, the ROS was elevated in a time-dependent manner (Figure 2(a)). The apoptosis protein was examined by western blotting, either. The results show that the antiapoptotic protein Bcl-2 was significantly decreased, and apoptotic protein caspase-9 was increased accordingly in a time-dependent manner (Figure 2(b)). It indicates that the apoptosis of the lung cancer cell coincided with elevated ROS. It has been reported that ROS scavenger N-acetylcysteine (NAC) could quench ROS and the subsequent apoptosis [4]. In truth, NAC in this study could reverse this LA-induced ROS (Figure 2(c)) and the corresponding proapoptotic effect (Figure 2(d)), indicating that LA could induce apoptosis also through the induction of ROS-apoptosis signaling.

### 2.3. Increment of ROS by the Degradation of NRF2 Contributes to the Proapoptosis of LA

**Figure 3:**
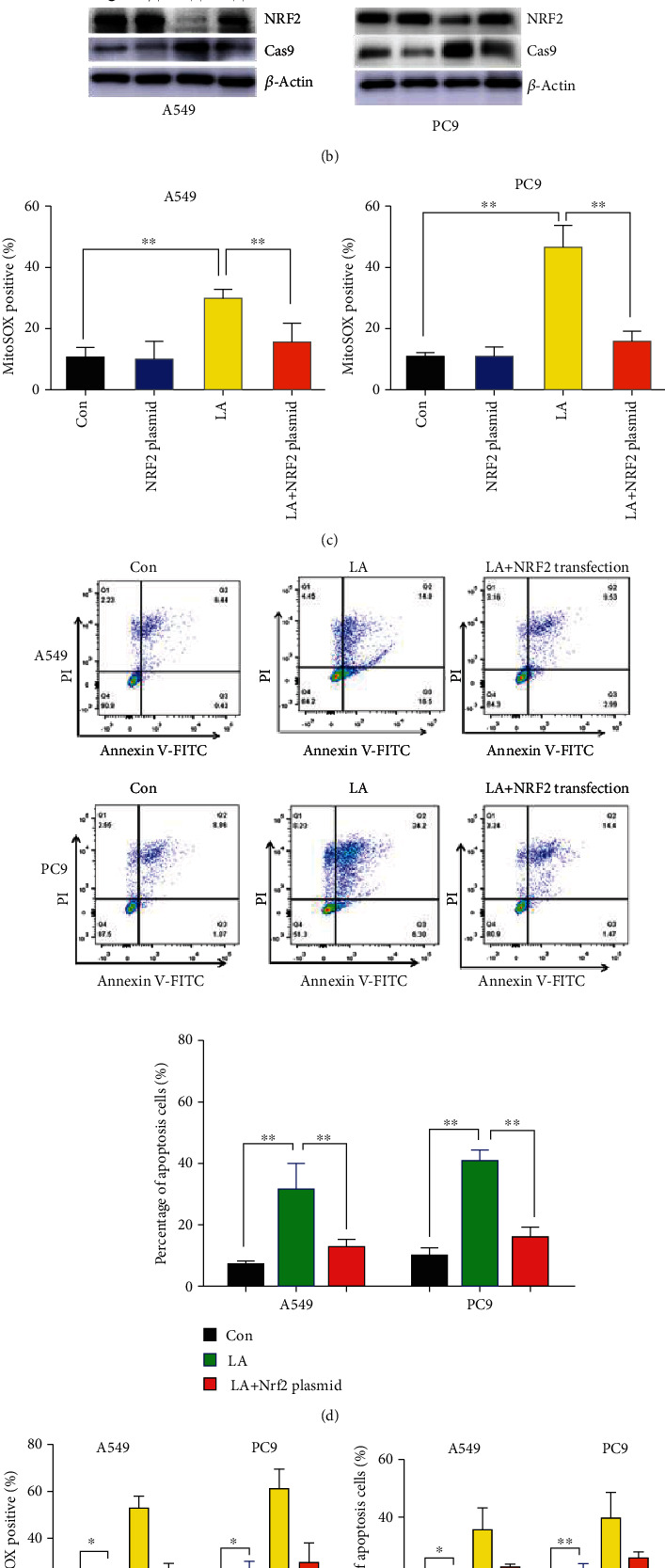
Downregulation of NRF2 by LA-induced apoptosis of A549 and PC9 cells. (a) NRF2 protein was downregulated in a dose- and time-dependent manner. (b) NRF2 and caspase-9 were examined in NRF2 expression plasmid transfected cells by western blotting. (c) Mitochondria ROS was measured by the MitoSOX red probe upon cells transfected with NRF2 expression plasmid after LA exposure. (d) NRF2 overexpression attenuated the LA-induced apoptosis in A549 and PC9 cells. (e) siRNA of NRF2-induced mitochondrial ROS and apoptosis. Results are shown as the mean ± s.d.∗∗ indicates *P* < 0.01.

Herein, the ROS leads to apoptosis upon LA exposure, so we hypothesized that LA induces apoptosis by downregulating the antioxidant system. Furthermore, as NRF2 has been broadly reported to keep redox homeostasis [25], it could control the antioxidant enzyme expression to battle the oxidative press. NRF2 protein levels of the two lung cancer cell lines were measured after LA exposure. As assumed, the protein levels of NRF2 were downregulated in a dose- and time-dependent manner (Figure 3(a)). To test if it is NRF2 downregulation that resulted in apoptosis, the NRF2 expression plasmid was transfected with cells after LA exposure. The results show that the downregulation of NRF2 protein could be elevated, and caspase-9 could be downregulated (Figure 3(b)). Also, the ROS and apoptosis induced by LA could be attenuated by NRF2 transfection (Figures 3(c) and 3(d)), indicating the apoptosis was negatively controlled by NRF2 expression. On the contrary, as LA could inhibit NRF2 leading to apoptosis, siRNA of NRF2 also results in elevated ROS and apoptosis (Figure 3(e)). All the data indicated that LA inhibits NRF2 to release ROS, initiating the apoptosis pathway.

### 2.4. LA Downregulates NRF2 by Suppressing PDK1 Expression

**Figure 4:**
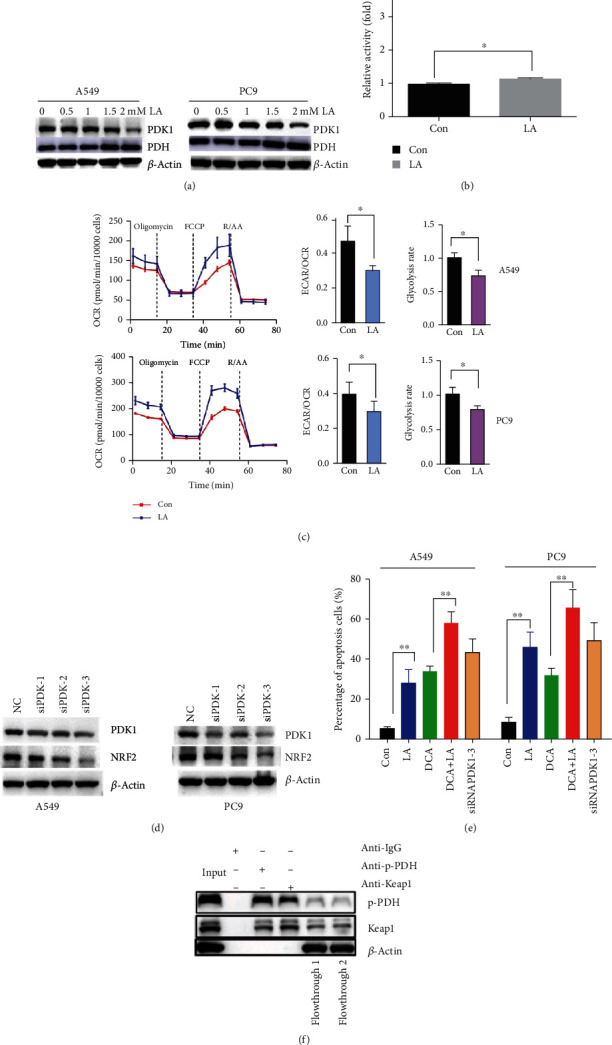
PDK1 inhibition by LA exerts proapoptotic effects through downregulating NRF2. (a) WB analysis of PDK1 and PDH expression in A549 and PC9 cells after LA exposure for 24 h. (b) LA (1.5 mM) slightly improves pyruvate dehydrogenase activity of A549 cells. (c) Glycolysis rate and oxygen consumption rate (OCR) were measured using glycolysis rate assay kit and Mito Stress Test kit. Calculated parameters of the assays are indicated in bar graphs. (d) Silencing of PDK1 by siRNA downregulates NRF2 expression. (e) PDK1 inhibition results in apoptosis and LA (1.5 mM) could enhance the proapoptosis effect of DCA (5 mM). Results are shown as the mean ± s.d.^∗^*P* < 0.05; ^∗∗^*P* < 0.01. (f) Endogenous coimmunoprecipitation between phosphorylated PDH and Keap1 in human lung cancer cells. A549 cells were collected and lysed to undergo Co-IP assay according to the manufacturer's protocol. After incubating with anti-P-PDH and anti-Keap1 beads, the elutes and flowthroughs (flowthrough 1 from anti-p-PDH beads; flowthrough 2 from anti-Keap beads) were checked by western blotting assay.

As LA acts as a cofactor of PDH, the latter switches the glycolysis to OXPHOS. According to the Warburg effect, most cancer cells tend to undergo glycolysis, including A549 and PC9 lung cancer cell lines [28], so we assume that LA influences the antioxidant system by regulating the mitochondrial metabolic enzymes. As assumed, PDK1 (the PDH kinase, which could phosphorylate PDH and rendering its inactivity), when processed with LA, could be inhibited in both the cell lines in a dose-dependent manner (Figure 4(a)). However, LA does not affect the total PDH protein level, and as expected, the PDH enzyme activity was slightly elevated (Figure 4(b)), which might be due to PDK1 inhibition. Furthermore, we measured glycolytic activity in real time by monitoring the extracellular acidification rate (ECAR) using the XF96 Seahorse apparatus (Figure 4(c)). Accordingly, we observed that basal glycolytic activity (glycolysis rate and the ratio of ECAR/OCR) was significantly reduced in A549 and PC9 cells upon LA exposure, which further confirmed PDK1 inhibition after LA exposure. Thus, we are curious about if there is some relationship between PDK1 inhibition and NRF2 downregulation.

By the siRNA-PDK1 interference technique, PDK1 downregulation was confirmed to inhibit NRF2 expression (Figure 4(d)). Of the three designed siRNAs, the third siRNA works best to inhibit PDK1 expression and inhibit NRF2 more robustly. This data indicated that LA downregulates PDK1 protein expression to inhibit NRF2. It has been checked that LA has no direct inhibitory effect to the purified PDK1 enzyme (data not shown) but just to PDK1 enzyme protein.

Interestingly, LA could coordinate with the well-known PDK1 inhibitor, dichloroacetate (DCA), which was reported inducing ROS and promote apoptosis of several cancer cells [29, 30]. Although LA inhibits lung cancer at high concentration with an average IC50 of about 3-6 mM, it enhances other antitumor agents such as DCA in the two lung cancer cells, indicating the clinical application prospects of LA (Figure 4(e)). Also, we noticed that siRNA PDK1 could result in apoptosis robustly (Figure 4(e)), indicating that PDK1 inhibition could lead to apoptosis.

As that PDK1 inhibition could downregulate NRF2 to promote apoptosis, it is necessary to know the mechanism of PDK1 inhibition regulating NRF2 inhibition. So, the interaction between phosphor-PDH (p-PDH) and the Keap1 (the specific NRF2 negative regulator) was tested by the co-IP technique, and the results showed that p-PDH could interact with Keap1 protein directly (Figure 4(f)). The antibody of human p-PDH or Keap1 was bound to beads. The cell lysis was incubated with the beads. After incubation, the flowthroughs were collected, the beads were washed, and the flowthrough and the elution were tested by western blotting for Keap1 and p-PDH binding separately. It is concluded that p-PDH would decrease upon PDK1 inhibition; then, the released Keap1 will result in NRF2 inhibition.

### 2.5. LA Inhibits Lung Carcinoma via Reducing PDK1 and NRF2 Expression *In Vivo*

**Figure 5:**
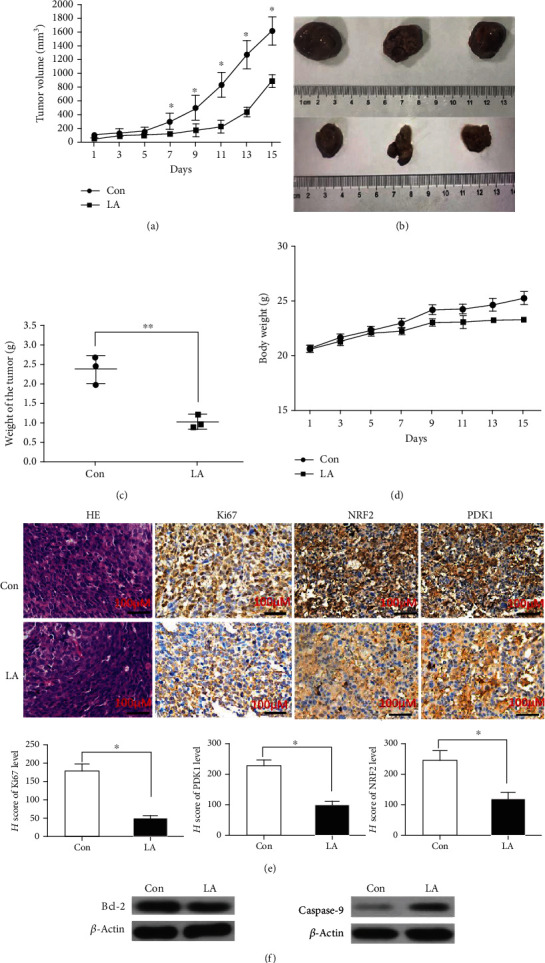
LA inhibits lung cancer growth and reduces NRF2 and PDK1 expression *in vivo*. (a) C57BL/6 mice bearing LLC tumor were treated with vehicle or LA (every other day by intraperitoneal injection) for 15 days, and then, tumor volume was recorded. It showed statistically significant differences in volume between the Con group and LA group (*P* < 0.05). (b) Image of tumors treated with LA or vehicle on day 16. (c) Comparison of tumor weight on day 16. (d) Effect of LA on mouse body weight. (e) The expression of patterns of Ki67, NRF2, and PDK1 was tested by immunohistochemical analysis in the tumors on day 16 in the control group and LA group. (f) Apoptotic protein Bcl-2 and caspase-9 expressions were analyzed in each tumor group. Results are shown as the mean ± s.d.∗ indicates *P* < 0.05; ^∗∗^*P* < 0.01.

To further demonstrate the anticancer effects of LA *in vivo* and the inhibition of NRF2 and PDK1, a tumor-bearing mouse (C57BL/6, black) model was constructed. Compared to the control group, intraperitoneal administration of LA (91 mg/kg) could significantly decrease the size (Figures 5(a) and 5(b)) and weight (Figure 5(c)) of the tumors formed by LLC cells (mouse lung cancer cells). In addition, the mice were tolerant to LA at the concentration used (Figure 5(d)). This could be due to the inhibition of cell proliferation and increase in cell apoptosis, which was revealed by a decrease in Ki67 staining (an antigen marker of cell proliferation which has usually been checked to indicate the proliferating ability of cancer cells) (Figure 5(e)) and decrease in Bcl-2 expression and, on the contrary, an increase in caspase-9 expression (Figure 5(f)). Consistent with the effect of LA on PDK1 and NRF2 inhibition *in vitro*, LA treatment also reduced the expression of PDK1 and NRF2 *in vivo* (Figure 5(e)). These results further support that LA could suppress lung cancer cell growth and promote apoptosis *in vivo*.

## 3. Discussion

Lung cancer is the most common primary cancer. Despite an increase in the 5-year survival rate over the past 40 years, no substantial improvement was seen since the 1980s [31, 32]. So, it is necessary to find novel therapeutic agents to combat this disease. In this study, we demonstrated that inducing degradation of NRF2 by inhibiting PDK1 is a promising strategy to combat lung cancer. LA (*α*-lipoic acid) is a novel PDK1 inhibitor for the treatment of lung cancer.

With the progression of cancer development, ROS rises with redox press. The strategy of cell adaptation to the redox press involves NRF2 to conquer the ROS press, and cell transformed for survival, striving for redox homeostasis. If ROS/apoptosis pathway should be activated, one should raise the ROS, but the NRF2 will combat the ROS. The metabolic will shift from oxidative phosphorylation to glycolysis leading to carcinogenesis [33]. An alternative strategy involves suppression of the antioxidant system, such as inhibition of NRF2 [34]. In this study, after LA exposure, the NRF2 was decreased, and ROS was increased.

In the past decades, LA has been reported the anticancer effects in broad aspects of cancer [16, 21]. Besides the anticancer, LA plays broad roles in physiological environment, including function in mitochondrial energy metabolism. LA is also known for its powerful antioxidative effects and has been used for the treatment of chronic diseases associated with high levels of oxidative stress, such as diabetic polyneuropathy and Alzheimer's disease. A growing number of studies have demonstrated that LA and its derivatives thereof are capable of suppressing the growth of various cancer cell lines, while nontransformed primary cells were hardly affected. The action mode of LA and its derivatives in cancer cells are described in detail, and promising results from recent preclinical studies are presented [35].

It seems a paradox that LA functions as both antioxidant and prooxidant. LA functions the prooxidant only in special cancer cells, such as A549 and PC9 cells which should show high-level NRF2 expression and high glycolytic level. In this study, through inhibiting PDK1 to further prohibit NRF2; LA functions as anticancer prooxidant. Moreover, LA exerted its antitumor effects at high concentration in this research work, which indicates potential antioxidant of LA at low dose and prooxidant of LA at high dose. The off-target effect of LA as anticancer prooxidant needs further study.

It could be noticed that the classic ROS generation pathway, such as PDH activity, showed slightly elevated (Figure 4(b)), indicating that a part of increased ROS, resulting from PDH activity improvement, but through the results of NRF2 overexpression, most of ROS generated by LA exposure could be ruined indicating that the induced ROS was due to NRF2 inhibition.

Many transactivation factors have been involved in the pathogenesis of cancer and were considered as drug targets. The agonist or antagonist is very difficult to develop as therapeutic targets [36]. Theoretically, the Keap1 binding to NRF2 will initiate the ubiquitin-proteasome degradation of NRF2. It was reported that the phosphorylation of Keap1 destroys the binding between NRF2 and Keap1 [7, 37]. It is very complicated to design small molecular inhibitors that target the phosphorylation process. A kind of ROS-inducing reagents that have proapoptotic effects assists in conquering cancer [38]. LA and DCA have been reported as ROS inducers with proapoptosis effect [23]. In this study, as a cofactor of PDH in the mitochondria, LA was found to be the PDK1 inhibitor and can decrease the transactivation factor NRF2, leading to the activation of the ROS/apoptosis pathway.

**Figure 6:**
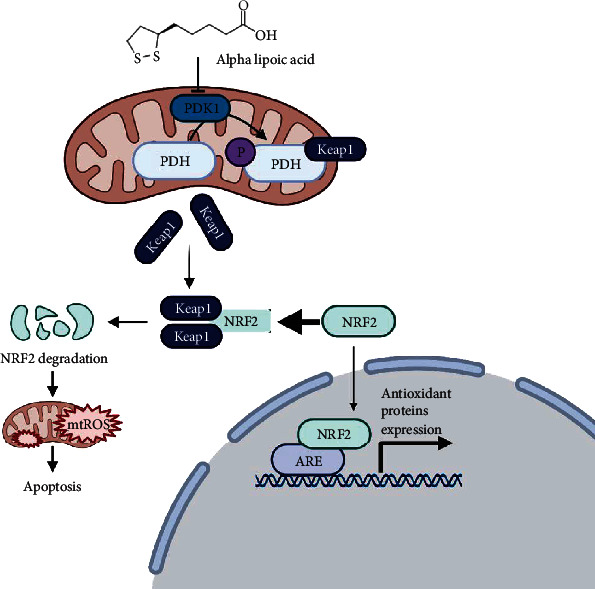
Schematic illustration of the apoptotic effect of LA in human lung cancer cells. LA inhibits PDK1, releasing Keap1 binded by p-PDH. NRF2 is then recaptured by Keap1 for degradation, initiating the ROS-induced apoptosis.

It is well known now that phosphorylation and nonphosphorylation state could play different roles [39]. PDK1, as a gatekeeping gene that assists in regulating the PDH activity, is the regulator for the PDH activity [33]. The phosphorylation state of PDH (p-PDH) broadly exists in the cancer cells. We speculate that phosphorylation of PDH serves as the regulator of the metabolism of energy and the redox homeostasis. In the study, we found that p-PDH can directly bind to Keap1, which is the negative controller of NRF2. Once PDK1 was inhibited, p-PDH (the product of PDK1) would be decreased, releasing the binding Keap1. Then, Keap1 is more feasible to destroy NRF2, thus elaborately regulating the ROS/apoptosis pathway. The detailed regulation process was summarized in the below scheme (Figure 6).

From the co-IP result, we find that the phosphorylated PDH could bind to Keap1. As many reported PDK1 inhibitors need large doses to induce ROS, also the apoptosis of cells seems to be reversible to grow well. We guess that the binding of p-PDH with Keap1 is a reversible process. The detailed mechanism still requires investigation. Nevertheless, how to develop powerful agents based on this result is still hopeful and exciting. When small doses were administered, LA functions as a cofactor of PDH to participate in pyruvate metabolism during cancer development. If it exceeds the physiologic threshold, it could function as an anticancer agent or cooperate with other proapoptosis reagents to function best. So, high doses of LA act as PDK1 inhibitor. On the other hand, PDK1 usually is little expressed in normal cells. So, LA is associated with meager side effects on clinical application. Also, for cancers that do not rely on PDK1 to hold switching between glycolysis and OXPHOS, the antitumor effects of LA still need to be investigated.

In summary, as a natural molecule, LA has a wide range of therapeutic effects, and it promotes apoptosis, showing fewer side effects. With a clearer molecular mechanism of LA proapoptosis, researchers can have a drug design scheme based on it to improve curative effect best.

## 4. Material and Methods

### 4.1. Reagents

LA and DCA were purchased from MedChemExpress（MCE, the USA）, and anti-human PDH, NRF2, Keap1, PDK1, and phosphor-PDH antibodies were purchased from the Cell Signaling Technology. Bcl-2, Caspase9 and *β*-actin antibodies were from Abcam. The LA and DCA were dissolved in dimethyl sulfoxide (DMSO). Dulbecco's modified Eagle's medium (DMEM) was obtained from Gibco. NAC (N-acetylcysteine) was purchased from SolarBio (Cat. No: IA0050).

### 4.2. Cell Culture

Human lung cancer cells A549 and PC9 (Shanghai Zhongqiao.com, Shanghai, China) were cultured in RPMI 1640 (Gibco) containing 10% FBS (Gibco) and 1% penicillin/streptomycin (Hyclone) in a 37°C, 5% CO_2_ incubator. Mouse cell line LLC (Shanghai Zhongqiao.com) was cultured in DMEM (Gibco, high glucose) containing 10% FBS (Gibco) and 1% penicillin/streptomycin (Hyclone) in a 37°C, 5% CO_2_ incubator.

### 4.3. Cell Viability Assay

Cell viability was detected using a CCK-8 kit (Cat. No. 40203ES60, Yeasen, Shanghai, China). Briefly, 5 × 10^3^ cells/well were seeded into 96-well plates, treated with different concentrations of LA, followed by addition of CCK-8 to each well at 24 h and 48 h. The optical density (OD) was measured after 2 h incubation at 37°C in an incubator. The data were processed using GraphPad Prism software by nonlinear regression curve fitting “log{inhibitor vs. response-variable slope four parameter”, and the formula was *Y* = Bottom + (Top − Bottom)/(1 + 10^((LogIC50 − *X*)∗HillSlope)^).

### 4.4. Flow Cytometry Analysis for Apoptosis

For apoptosis analysis, A549 and PC9 cells were treated with different concentration of LA for 24 h and 48 h, washed with cold PBS, and then collected. A mixture of Annexin V-FITC/PI (Cat. No. 556547, BD, San Jose, CA) was added. The cells were then analyzed by flow cytometry (BD Biosciences, USA) immediately after incubation at room temperature for 20 min in the dark.

### 4.5. Flow Cytometry Analysis for Mitochondrial ROS

For MitoSOX staining, cells were incubated in phenol red-free medium containing 10% serum and 2.5 *μ*M MitoSOX Red (Cat. No. M36008, Thermo Fisher Scientific) at 37°C for 10 min. Cells were then trypsinized, rinsed once with cold PBS, and resuspended in PBS supplemented with 0.1% serum. MitoSOX samples were immediately analyzed using a BD FACSCanto II flow cytometry system (BD Biosciences). For gating, cells were first gated by size using FSC and SSC and then by FSC height × FSC area to exclude doublets before measuring the fluorescence. Universally, the intensity threshold was set by which there are 10% positive cells of the control sample (without LA). The cell with signal beyond the threshold was regarded as the MitoSOX-positive cells, using the positive percentage to make the bar graphs. The MitoSOX quantification methods were done according to the reported methods [11].

### 4.6. siRNA Analysis

Human PDK1 siRNA and NRF2 siRNA (Hanheng Biotechnology, Shanghai, China) were used to investigate PDK1 and NRF2 expression profiles. The fragmented RNA was loaded onto the cells to operate according to the product protocol.

### 4.7. Cell Transfection

A549 or PC9 lung cancer cells were seeded in culture plates in serum-free DMEM. Three siRNAs (siRNA-1, siRNA-2, and siRNA-3) were designed and synthesized targeting PDK1, as well as a nonspecific siRNA (NC) and a GAPDH siRNA (siRNA-NC), and then were transfected into separate groups of A549 or PC9 lung cancer cells. siRNA-3 was evaluated as the most effective PDK1 siRNA. The sequences of the three PDK1 siRNAs were siRNA-1: 5′-GGAUCAGAAACCGACACAATT-3′; siRNA-2: 5′-GCCAAUACAAGUGGUUUAUTT-3′; and siRNA-3: 5′-GCUAGGCGUCUGUGUGAUUTT-3′; 6 h after transfection, fresh medium was added to the transfected cells and incubated for 24 h. Finally, verifying the gene silence is by determining the expression of proteins using western blot. The sequences of the three NRF2 siRNAs were siRNA-1: 5′-GGUUGAGACUACCAUGGUUTT-3′; siRNA-2: 5′-CCUGAAAGCACAGCAGAAUTT-3′; and siRNA-3: 5′-GCAGUUCAAUGAAGCUCAATT-3′.

### 4.8. NRF2 Overexpression

The plasmids of NRF2 were obtained from Hanheng Biotechnology. The cells with or without LA exposure were mixed with the transfection mixture (Lipofectamine 2000 with vehicle vector or NRF2 plasmid). After six hours of incubation, the cells were rinsed and incubated for another 24 h before the following analysis.

### 4.9. Western Blotting Analysis

The pretreated cells were washed with cold PBS. The concentration and purity of total cellular proteins were determined after being lysed on ice in RIPA buffer containing the protease inhibitor. An equal amount of protein was then loaded onto a 10% or 12.5% SDS-PAGE gel, transferred to nitrocellulose membranes, and incubated with primary antibodies overnight at 4°C after blocking for 1 h with 3% BSA, using anti-p-PDH, anti-PDH, anti-PDK1, anti-NRF2, anti-Keap1, anti-Bcl-2, anti-caspase-9, and anti-*β*-actin (1 : 500). After three washes with PBST, membranes were incubated with the HRP-conjugated secondary antibodies, and then visualized by chemiluminescence.

### 4.10. PDH Activity Assay

After processing with or without LA for 24 h, the A549 and PC9 cells were processed according to the PDH activity assay kit for measuring the PDH activity. The kit was purchased from Sigma (Cat. No MAK183).

### 4.11. Extracellular Flux Analysis

A549 and PC9 cells were plated at 7500 cells/well in XF96 microplates (Seahorse Bioscience, Billerica, MA, USA) in RPMI medium with 10% according to the manufacturer's recommendations 48 h prior to the assay. Treatment with 1.5 mM LA was performed for 24 h.

For Mito Stress Test, the medium was exchanged to XF base medium (Seahorse Bioscience, Billerica, MA, USA) with 1 mM pyruvate, 2 mM glutamine, and 10 mM glucose and incubated at 37°C without CO_2_ 1 h prior to the assay. During the assay, OCR was measured using a Seahorse XFe 96 analyzer. Mito Stress Test kit containing 1 *μ*M oligomycin, 1 *μ*M carbonyl cyanide-4-(trifluoromethoxy)phenylhydrazone (FCCP), and 0.5 *μ*M rotenone/0.5 *μ*M antimycin A was performed according to the manufacturer's protocol.

For glycolytic rate assay, the medium was exchanged to XF base medium without phenol red (Seahorse Bioscience, Billerica, MA, USA) with 2 mM glutamine, 10 mM glucose, 1 mM pyruvate, and 5 mM HEPES and incubated at 37°C without CO_2_ 1 h prior to the assay. During the assay, ECAR was measured using a Seahorse XFe 96 analyzer. Glycolytic rate assay kit containing 0.5 *μ*M rotenone, 0.5 *μ*M antimycin A, and 50 mM 2-desoxyglucose was performed according to the manufacturer's protocol. ECAR and OCR were calculated by Wave 2.6 (Seahorse Bioscience, Billerica, MA, USA) software after the assay and normalized to cell numbers.

### 4.12. Coimmunoprecipitation (Co-IP) Assay

The A549 cells were lysed in an immunoprecipitation lysis buffer containing a protease inhibitor and phosphatase inhibitor. After centrifugation, the collected supernatant was incubated with beads by covalent binding antibody (the coimmunoprecipitation kit was purchased from Thermo Fisher, Cat. No. 26149) at 4°C overnight. The beads were then rinsed, and the bound proteins were eluted and then evaluated using western blotting analysis.

### 4.13. Tumor-Bearing Model Construction

Male C57BL/6 black mice aged six weeks (Shanghai Laboratory Animal Center, Shanghai, China) were fed with a standard diet and had free access to water in the standard animal laboratory. After subcutaneous injection of 100 *μ*l LLC mouse lung cancer cells (3 × 106, serum-free culture medium) into the abdomen of mice, 6 animals were equally and randomly divided into two groups: a control group (ordinary diet) and an LA group (91 mg/kg LA, abdominal administration for three times a week) and sacrificed 15 days after administration. The tumors were removed, paraffin-embedded, fixed in 4% paraformaldehyde for 24 h, and then sliced into 5 *μ*m thick sections for immunohistochemical analysis.

### 4.14. Histology and Immunohistochemistry

Tumors were cut into 2 *μ*m sections from at least 3 different planes and stained with haematoxylin and eosin. For Ki67, NRF2, and PDK1 immunoperoxidase staining, paraffin-embedded sections were dehydrated and antigenic epitopes were exposed using a 10 mM citrate buffer and microwaving. Sections were incubated with rabbit polyclonal anti-Ki67, anti-NRF2, and anti-PDK1 antibodies. Primary Ab staining was detected by peroxidase-conjugated anti-rabbit IgG. The images were acquired under a Leica DM 4000B fluorescence microscope equipped with a digital camera. For the quantitative analysis, a histoscore (*H* score) was calculated based on the staining intensity and percentage of stained cells. The intensity score was defined as such: 0, no appreciable staining in cells; 1, weak staining in cells comparable with stromal cells; 2, intermediate staining; and 3, strong staining. The fraction of positive cells was scored as 0–100%. The *H* score was calculated by multiplying the intensity score and the fraction score, producing a total range of 0–300. A cutoff of 30 was used for LA positivity. Tissue sections were examined and scored separately by two independent investigators blinded to the clinicopathologic data.

### 4.15. Statistical Analysis

The GraphPad was used for statistical analysis, and all the data were obtained from at least three independent experiments. The results were calculated using Student's *t*-test and one-way ANOVA. ^∗^*P* < 0.05 and ^∗∗^*P* < 0.01, compared to the control group (0.5% DMSO).

## Data Availability

The data used to support the findings of this study are available from the corresponding authors upon request.

## References

[1] Reuter S., Gupta S. C., Chaturvedi M. M., Aggarwal B. B. (2010). Oxidative stress, inflammation, and cancer: how are they linked?. *Free Radical Biology & Medicine*.

[2] Visconti R., Grieco D. (2009). New insights on oxidative stress in cancer. *Current Opinion in Drug Discovery & Development*.

[3] Waris G., Ahsan H. (2006). Reactive oxygen species: role in the development of cancer and various chronic conditions. *Journal of Carcinogenesis*.

[4] Moungjaroen J., Nimmannit U., Callery P. S. (2006). Reactive oxygen species mediate caspase activation and apoptosis induced by lipoic acid in human lung epithelial cancer cells through Bcl-2 down-regulation. *The Journal of Pharmacology and Experimental Therapeutics*.

[5] Huang P., Feng L., Oldham E. A., Keating M. J., Plunkett W. (2000). Superoxide dismutase as a target for the selective killing of cancer cells. *Nature*.

[6] Trachootham D., Alexandre J., Huang P. (2009). Targeting cancer cells by ROS-mediated mechanisms: a radical therapeutic approach?. *Nature Reviews. Drug Discovery*.

[7] Krall E. B., Wang B., Munoz D. M. (2017). Correction:KEAP1 loss modulates sensitivity to kinase targeted therapy in lung cancer. *eLife*.

[8] Liu P., Tian W., Tao S. (2019). Non-covalent NRF2 activation confers greater cellular protection than covalent activation. *Cell Chemical Biology*.

[9] Swamy S. M., Rajasekaran N. S., Thannickal V. J. (2016). Nuclear factor-erythroid-2-related factor 2 in aging and lung fibrosis. *The American Journal of Pathology*.

[10] Hlouschek J. R. V., Ritter V., Wirsdörfer F., Klein D., Jendrossek V., Matschke J. (2018). Targeting SLC25A10 alleviates improved antioxidant capacity and associated radioresistance of cancer cells induced by chronic-cycling hypoxia. *Cancer Letters*.

[11] Fox D. B., Garcia N. M. G., McKinney B. J. (2020). NRF2 activation promotes the recurrence of dormant tumour cells through regulation of redox and nucleotide metabolism. *Nature Metabolism*.

[12] Kaneda M. M., Messer K. S., Ralainirina N. (2016). PI3K*γ* is a molecular switch that controls immune suppression. *Nature*.

[13] Taniguchi S., Elhance A., van Duzer A., Kumar S., Leitenberger J. J., Oshimori N. (2020). Tumor-initiating cells establish an IL-33–TGF-*β* niche signaling loop to promote cancer progression. *Science*.

[14] Wang H., Gao Z., Liu X. (2018). Targeted production of reactive oxygen species in mitochondria to overcome cancer drug resistance. *Nature Communications*.

[15] Gao Ting X. Y., Xiaoying Z., Zhong C., Jianping Y. (2018). Mechanism of regulation of cell energy metabolism by mitochondrial lipoic acid synthesis. *Chinese Journal of Cell Biology*.

[16] Dörsam B., Fahrer J. (2016). The disulfide compound *α*-lipoic acid and its derivatives: a novel class of anticancer agents targeting mitochondria. *Cancer Letters*.

[17] Liu J. (2008). The effects and mechanisms of mitochondrial nutrient *α*-lipoic acid on improving age-associated mitochondrial and cognitive dysfunction: an overview. *Neurochemical Research*.

[18] Guo Y., Qiu W., Roche T. E., Hackert M. L. (2020). Crystal structure of the catalytic subunit of bovine pyruvate dehydrogenase phosphatase. *Acta Cryst*.

[19] Phiboonchaiyanan P., Chanvorachote P. (2017). Suppression of a cancer stem-like phenotype mediated by alpha-lipoic acid in human lung cancer cells through down-regulation of *β*-catenin and Oct-4. *Cellular Oncology*.

[20] Cimolai M. C., Vanasco V., Marchini T., Magnani N. D., Evelson P., Alvarez S. (2014). *α*-Lipoic acid protects kidney from oxidative stress and mitochondrial dysfunction associated to inflammatory conditions. *Food &Function*.

[21] Park S., Choi S. K., Choi Y., Moon H. S. (2015). AMPK/p53 axis is essential for *α*-lipoic acid–regulated metastasis in human and mouse colon cancer cells. *Journal of Investigative Medicine*.

[22] Kim S. J., Kim H. S., Seo Y. R. (2019). Understanding of ROS-inducing strategy in anticancer therapy. *Oxidative Medicine and Cellular Longevity*.

[23] Yan X.-J., Xie P., Dai X.-F. (2020). Dichloroacetate enhances the antitumor effect of pirarubicin via regulating the ROS-JNK signaling pathway in liver cancer cells. *Cancer Drug Resistance*.

[24] Pedersen P. L. (1978). Tumor mitochondria and the bioenergetics of cancer cells. *Progress in Experimental Tumor Research*.

[25] Seifert F., Ciszak E., Korotchkina L. (2007). Phosphorylation of serine 264 impedes active site accessibility in the E1 component of the human pyruvate dehydrogenase multienzyme complex. *Biochemistry*.

[26] Deng X., Wang Q., Cheng M. (2020). Pyruvate dehydrogenase kinase 1 interferes with glucose metabolism reprogramming and mitochondrial quality control to aggravate stress damage in cancer. *Journal of Cancer*.

[27] Sun W., Xie Z., Liu Y. (2015). JX06 selectively inhibits pyruvate dehydrogenase kinase PDK1 by a covalent cysteine modification. *Cancer Research*.

[28] Bao J., Yan W., Xu K. (2020). Oleanolic acid decreases IL-1*β*-induced activation of fibroblast-like synoviocytes via the SIRT3-NF-*κ*B axis in osteoarthritis. *Oxidative Medicine and Cellular Longevity*.

[29] Li B., Zhu Y., Sun Q. (2018). Reversal of the Warburg effect with DCA in PDGF‑treated human PASMC is potentiated by pyruvate dehydrogenase kinase‑1 inhibition mediated through blocking Akt/GSK‑3*β* signalling. *International Journal of Molecular Medicine*.

[30] Peng F., Wang J. H., Fan W. J. (2018). Glycolysis gatekeeper PDK1 reprograms breast cancer stem cells under hypoxia. *Oncogene*.

[31] Heist R. S., Engelman J. A. (2012). SnapShot: non-small cell lung cancer. *Cancer Cell*.

[32] Warth A. (2015). Diagnose, prognose und prädiktion nicht-kleinzelliger lungenkarzinome. *Der Pathologe*.

[33] Kaplon J., Zheng L., Meissl K. (2013). A key role for mitochondrial gatekeeper pyruvate dehydrogenase in oncogene-induced senescence. *Nature*.

[34] Dodson M., de la Vega M. R., Cholanians A. B., Schmidlin C. J., Chapman E., Zhang D. D. (2019). Modulating NRF2 in disease: timing is everything. *Annual Review of Pharmacology and Toxicology*.

[35] Tibullo D., Li Volti G., Giallongo C. (2017). Biochemical and clinical relevance of alpha lipoic acid: antioxidant and anti-inflammatory activity, molecular pathways and therapeutic potential. *Inflammation Research*.

[36] Cosimelli B., Greco G., Laneri S. (2019). Identification of novel indole derivatives acting as inhibitors of the Keap1-Nrf2 interaction. *Journal of Enzyme Inhibition and Medicinal Chemistry*.

[37] Bollong M. J., Lee G., Coukos J. S. (2018). A metabolite-derived protein modification integrates glycolysis with KEAP1-NRF2 signalling. *Nature*.

[38] Lastra D., Fernandez-Gines R., Manda G., Cuadrado A. (2021). Perspectives on the clinical development of NRF2-targeting drugs. *Handbook of Experimental Pharmacology*.

[39] Zhang W., Su J., Xu H. (2017). Dicumarol inhibits PDK1 and targets multiple malignant behaviors of ovarian cancer cells. *PLoS One*.

